# Levothyroxine Treatment and the Risk of Cardiac Arrhythmias – Focus on the Patient Submitted to Thyroid Surgery

**DOI:** 10.3389/fendo.2021.758043

**Published:** 2021-11-04

**Authors:** Zoran Gluvic, Milan Obradovic, Alan J. Stewart, Magbubah Essack, Samantha J. Pitt, Vladimir Samardzic, Sanja Soskic, Takashi Gojobori, Esma R. Isenovic

**Affiliations:** ^1^Clinic for Internal Medicine, Department of Endocrinology and Diabetes, Zemun Clinical Hospital, School of Medicine, University of Belgrade, Belgrade, Serbia; ^2^Department of Radiobiology and Molecular Genetics, VINČA Institute of Nuclear Sciences - National Institute of the Republic of Serbia, University of Belgrade, Belgrade, Serbia; ^3^School of Medicine, University of St Andrews, St Andrews, United Kingdom; ^4^King Abdullah University of Science and Technology (KAUST), Computer, Electrical, and Mathematical Sciences and Engineering (CEMSE) Division, Computational Bioscience Research Center (CBRC), Thuwal, Saudi Arabia

**Keywords:** levothyroxine, thyroid, hypothyroidism, cardiac arrhythmias, osteoporosis, replacement therapy

## Abstract

Levothyroxine (LT4) is used to treat frequently encountered endocrinopathies such as thyroid diseases. It is regularly used in clinical (overt) hypothyroidism cases and subclinical (latent) hypothyroidism cases in the last decade. Suppressive LT4 therapy is also part of the medical regimen used to manage thyroid malignancies after a thyroidectomy. LT4 treatment possesses dual effects: substituting new-onset thyroid hormone deficiency and suppressing the local and distant malignancy spreading in cancer. It is the practice to administer LT4 in less-than-high suppressive doses for growth control of thyroid nodules and goiter, even in patients with preserved thyroid function. Despite its approved safety for clinical use, LT4 can sometimes induce side-effects, more often recorded with patients under treatment with LT4 suppressive doses than in unintentionally LT4-overdosed patients. Cardiac arrhythmias and the deterioration of osteoporosis are the most frequently documented side-effects of LT4 therapy. It also lowers the threshold for the onset or aggravation of cardiac arrhythmias for patients with pre-existing heart diseases. To improve the quality of life in LT4-substituted patients, clinicians often prescribe higher doses of LT4 to reach low normal TSH levels to achieve cellular euthyroidism. In such circumstances, the risk of cardiac arrhythmias, particularly atrial fibrillation, increases, and the combined use of LT4 and triiodothyronine further complicates such risk. This review summarizes the relevant available data related to LT4 suppressive treatment and the associated risk of cardiac arrhythmia.

## 1 Introduction

Thyroidectomy is a surgical procedure, performed either as a standard open surgery or as an alternative approach surgery, such as minimally invasive video-assisted thyroidectomy (MIVAT) or robot-assisted transaxillary thyroidectomy, aiming to remove all or part of the thyroid gland (1). The procedure is commonly used to treat a range of thyroid-related disorders, including thyroid cancer, hyperthyroidism goiters, and thyroid nodules that can be obstructive and cause swallowing or breathing difficulties (2). The introduction of MIVAT improved the treatment options for some thyroid conditions. Despite superiority regarding patients’ satisfaction with faster recovery and decreased complications associated with standard open thyroidectomy (neck pain, voice problems, anxiety), it is confirmed as a reliable procedure in only strictly indicated cases (1). It is not suitable for patients with thyroiditis, large multinodular goiters, locally invasive thyroid carcinoma, or the presence of lateral neck compartment malignant lymph nodes. It evolves as standard procedure in the carefully selected cases with low- and intermediate-risk differentiated thyroid carcinoma (3, 4).

The thyroid gland produces the iodine-containing thyroid hormones, triiodothyronine (T3) and thyroxine (T4) in response to thyroid stimulation hormone (TSH) and the peptide hormone calcitonin, which is primarily regulated by serum calcium levels (5, 6). Together, these hormones regulate a wide range of metabolic and cardiovascular processes, including basal metabolic rate, appetite, gut motility, nutrient absorption, rate and strength of heart contractions, breathing, and oxygen consumption (7). Thyroid hormones also play a developmental role; they are essential for cell growth, while cells of the developing brain are a major target for T3 and T4 (8).

When the whole thyroid is extirpated, such gland surgery is referred to as total thyroidectomy. Knowing that thyroid hormones are essential for life, it is necessary to permanently replace the resultant deficiency with thyroxine after total thyroidectomy. Without replacement, a patient will develop signs and symptoms of hypothyroidism. Standard treatment in such instances is the long-term prescription of the synthetic thyroid hormone levothyroxine (LT4, a manufactured form of T4). In cancer cases, LT4 treatment after thyroidectomy can have the added advantage of suppressing local and distant malignancies from spreading. However, the LT4 dose must be carefully optimized to avoid potential adverse effects such as weight loss, sweating, anxiety, insomnia, osteoporosis (increased bone fracture risk), and an increased heart rate. Thus, LT4 treatment in individuals that have suffered a recent heart attack is cautiously recommended (9). It is not surprising that some individuals experience cardiovascular complications following LT4 treatment as thyroid hormones regulate cardiac functioning. Indeed, increased thyroid hormone levels are associated with an increased risk of developing heart arrhythmias (10). Here we examine the relevant literature related to LT4 treatment after thyroidectomy and the associated risk of cardiac arrhythmias in such patients.

## 2 Levothyroxine (LT4): Structure, Brief History, Pharmacokinetics, Pharmacodynamics, Dosing Regimens

**Figure 1:**
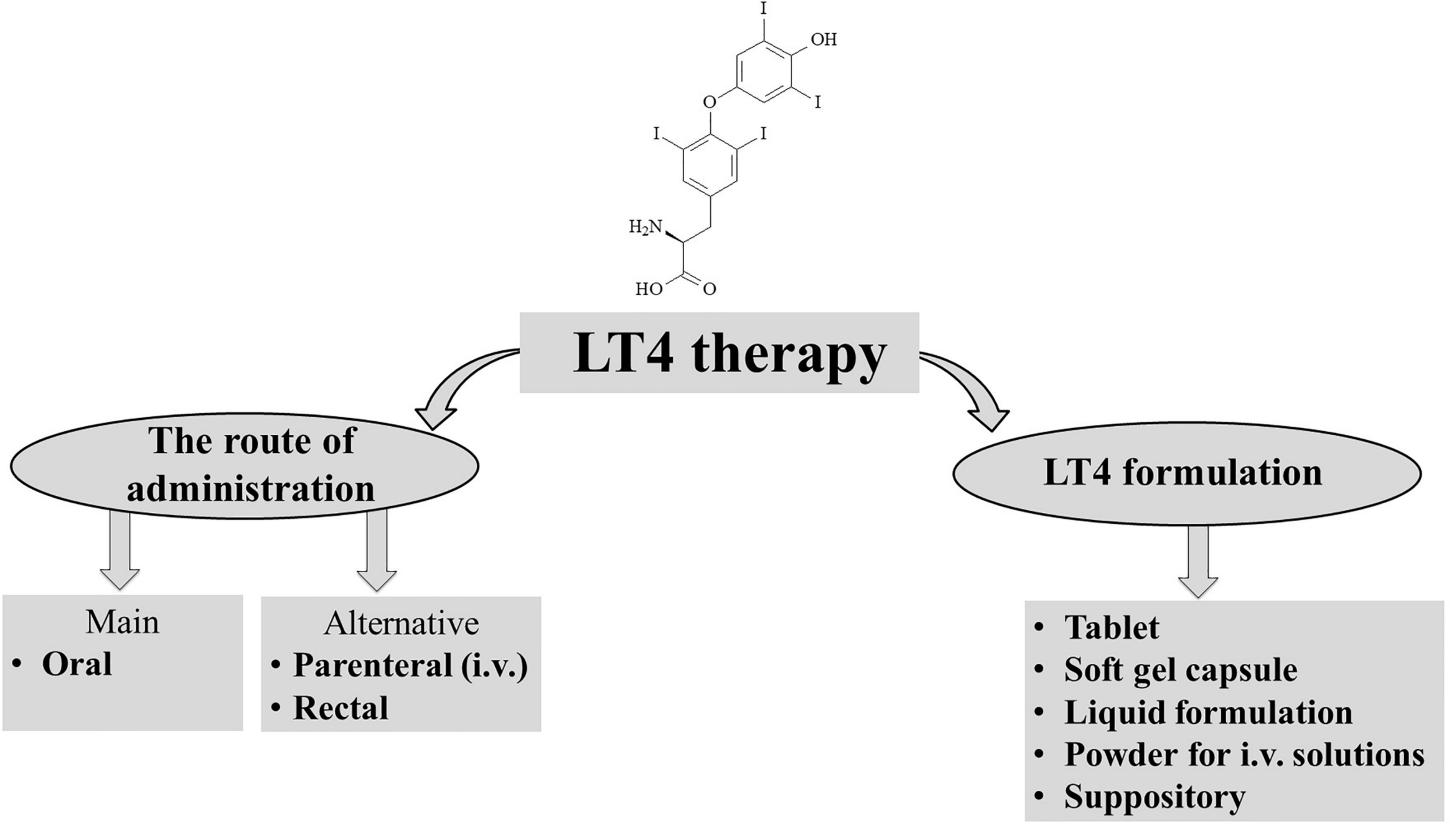
Levothyroxin (LT4) therapy.

Levothyroxine is a synthetic version of the secreted thyroid hormone thyroxine (T4) that completely mimics all physiologic effects of T4 (**Figure 1**). LT4 is used as replacement therapy in primary-thyroidal, secondary-pituitary, and tertiary-hypothalamic hypothyroidism (11, 12). Despite T4 being naturally present as a racemic mixture of the levo and dextro forms, LT4 is produced as a levo-isomer due to its greater physiological activity than the dextro form (13, 14).

The use of LT4 as a standard monotherapy came to the fore in the 1970s with evidence that T3 is predominantly produced by peripheral deiodination of T4. In patients treated with LT4 alone, thyroid function becomes normalized (15–17). Before that, combination therapy of synthetic LT4 and LT3 was the standard hormone replacement therapy in hypothyroid patients (18). Hypothyroidism treatment dates from the 6th century and Chinese medicine, where animal thyroid was used for therapy (17, 19). The same approach was applied in Europe but later in the 19^th^ century (17, 19). In the 20^th^ century, the discovery of thyroid hormones accelerated progress towards developing the current therapies (13, 17).

Adults with newly diagnosed hypothyroidism and without other complications receive an initial dose of 1.6 µg/kg/day for a few months. After dose modification, it is recommended to check the TSH level every 6-8 weeks (11, 12). In adult hypothyroid patients, hypothyroid patients with pre-existing heart diseases or >65 years old, an initial dose of 25 µg/day of LT4 is given, followed by an adjusted dose of 12.5 to 25 mcg every 4-6 weeks (11, 12). In patients with severe hypothyroidism or myxedema coma, the initial dose may be 200 to 400 µg administered *via* nasogastric tube or intravenously, followed by a daily dose of 1.2 µg/kg/day. Older patients or patients with heart disease are recommended to use lower doses (11, 12). It is recommended to decrease the LT4 dose for elderly patients. Regarding suppressive treatment for the control of thyroid nodule growth, the LT4 dose is individually tailored to maintain TSH levels as low-normal or at a partial suppressive level. In the high-risk patients following thyroidectomy for well-differentiated thyroid cancer, the estimated LT4 full suppressive dose is ≥2 µg/kg body weight (11, 12, 20–23).

The level of thyroid-stimulating hormone (TSH) in serum is used as an indicator of monitoring and adjusting the dose of LT4 therapy in hypothyroid patients, except in patients with secondary or tertiary hypothyroidism, where the level of free or total T4 is used as a marker for the success of the therapy (11, 12, 24). The standard monitoring procedure requires determining TSH levels 6-8 weeks after the initial treatment with LT4 (11, 12). After achieving the correct dose of LT4, the level of TSH is monitored firstly at 4-6 months, and after that, every 12 months (11, 12).

LT4 toxicity is rare, but adverse effects due to inappropriate dosage (over-or under-dose) can occur, especially in patients with pre-existing comorbidities such as cardiovascular disease, uncorrected adrenal insufficiency, and elderly patients (25).

Absorption of orally administered LT4 from the gastrointestinal tract mainly occurs in the small intestine rather than the stomach (26, 27). The absorbed LT4 varies from 60% to 80%, with the maximum concentration in circulation achieved 3 hours after administration in hypothyroid subjects and slightly faster in euthyroid subjects, approximately 2 hours (28–30). Several factors influence LT4 absorption, including deranged small intestine physiology (e.g. bowel resection reduced absorption), fasting increased absorption, while different foods, drugs, and supplements can also disturb LT4 absorption (30, 31). All these factors indicate the need for permanent monitoring in individual approaches in LT4 replacement (32). The half-life for T_4_ is ~7.5 days in patients with primary hypothyroidism, with a daily turnover rate of ~10% for T_4_ and 50–70% for T_3_ (33). Contrary, in euthyroid subjects, the half-life for T4 is 6.2 days, and a little faster turnover rate (34–36). In addition, the estimated T_3_ half-life is 1.4 days in hypothyroid patients and 1.0 days for euthyroid individuals (34–36). The values reported for T4 clearance are very close in hypothyroid subjects (approx. 0.04-0.06 l/h) and normal control individuals (0.05-0.06 l/h) (33, 37).

The liver is the primary site of LT4 degradation (38, 39). Although T_4_ is catabolized *via* several routes, the major pathway of T_4_ catabolism is sequential deiodination in the presence of deiodinase enzymes (38–40). The removal of iodine from carbon 5 of the outer ring of T4 converts it to T3, while removing iodine from the inner ring of T4 leads to the formation of inactive reverse T3 (rT3) (41, 42). T3 and rT3 originate from T4 at an ~1:1 ratio, and about 80% of T3 in circulation stems from peripheral T4 (43, 44). Subsequently, T3 can be converted to both diiodothyronine (T2) and iodothyronine (T1), and rT3 to both rT2 and rT1 (45, 46).

## 3 Thyroid Diseases and Conditions Associated With LT4 Use

Long-term use of levothyroxine (LT4) has been demonstrated to be effective and safe. Initially, LT4 was used only in thyroxin (T4) deficiency cases, but LT4 usage has evolved and includes substitution and suppressive therapy (17). The aim of LT4 treatment differs according to the indication of use. In hypothyroid subjects, the goal of LT4 substitution is to establish a euthyroid rank of TSH while improving quality of life. The goal of LT4 treatment in controlling nodule growth is a low normal TSH level but avoiding LT4 overdose (47). After surgical management of malignant disease, treatment goals are to obtain suppressed levels of both TSH and thyroglobulin (48). Lower doses of LT4 are necessary to achieve substitution goals in elderly patients, while higher LT4 doses are required in patients undergoing total thyroidectomy (49). Long-term use of LT4 substitution reduces the risk of bradycardia, which is often associated with hypothyroidism (50). In patients with low-normal or suppressed TSH levels, an increased risk of cardiac arrhythmias, primarily atrial fibrillation (AF), is observed (10, 51, 52), as is an increased risk of osteoporosis (53, 54) and overall mortality (55). However, Flynn et al. (52) showed that patients with TSH levels between 0.04 to 0.4 mIU/ml did not experience an increased risk of cardiovascular disease, arrhythmias, or osteoporotic fractures (56).

The management with LT4 after MIVAT, either substitutive or suppressive modality, depends on the pathology of the partially or entirely extirpated thyroid gland. Each LT4 management modality must be individually tailored to the patient. MIVAT treated benign diseases require standard LT4 substitution therapy, while malignant disease (i.e. differentiated thyroid cancer) requires partial or complete TSH suppression depending on assigned risk. In high-risk patients, a complete suppressive LT4 regimen (TSH <0.1 mIU/ml) is recommended opposite to a partial LT4 suppressive regimen in lower-risk patients (TSH 0.1-0.4 mIU/ml) (48, 57).

In addition to hypothyroidism classification according to clinical presentation (subclinical or latent and clinical or overt), there is another classification based on the level of lesion-induced thyroid dysfunction. Primary hypothyroidism is the most frequently encountered in clinical practice (49). Secondary (at pituitary level) hypothyroidism, tertiary (at hypothalamus level) hypothyroidism, and thyroid hormone resistance account for less than 1% of overall hypothyroidism. They are mostly presented with symptoms and signs of mild hypothyroidism and sometimes with local effects (i.e., symptoms and signs of increased intracranial pressure). Besides the clinical presentation of hypothyroidism, thyroid hormone resistance syndromes can also present as mental health problems (49, 58, 59). LT4-dose tapering is more complex in patients with secondary and tertiary hypothyroidism as the fT4 levels, which is the basis of how the quality of LT4 dosing is assessed, are less flexible (49, 60). Whether iatrogenic or disease-induced, all forms of hypothyroidism (subclinical or clinical) unequivocally accelerate atherosclerosis processes, which can contribute to increased cardiovascular morbidity and mortality (61–66).

The prevalence of overt hypothyroidism in the general population is 0.3–3.7% in the USA (67) and 0.2–5.3% in Europe (68) depending on the definition of hypothyroidism. Hypothyroidism is more common in people over 65 years, women, and Caucasians. Among these, the most common cause of primary hypothyroidism in iodine-sufficient areas is chronic autoimmune thyroiditis. Although thyroid anti-peroxidase antibodies are of diagnostic significance, they also present in about 11% of people with no thyroid disease (49, 69).

## 4 LT4 Replacement Therapy in Cardiovascular Patients

LT4 replacement therapy compensates for endogenous thyroxine deficiency, whether the disease’s latent (subclinical) or manifested (clinical) form or post-procedural hypothyroidism. Excessive LT4 substitution in cardiovascular patients can have serious side effects, such as AF and osteoporosis, especially in postmenopausal women and elderly patients (49, 52). These complications are frequently observed in Hashimoto’s thyroiditis patients due to LT4 over-supplementation (54). In cases of long-term TSH suppression, an increase in left ventricular mass and consequent diastolic dysfunction may occur, which further contributes to cardiovascular morbidity, especially in those patients with already diagnosed cardiovascular disease (70). A study by Petersen et al. (71), suggested that the prevalence of ischemic heart disease in people over 65 years of age with suppressed TSH levels on LT4 substitution was increased relative to the general population. Other studies suggest that atherosclerosis acceleration can occur during subclinical hypothyroidism development (72, 73).

It is known that thyroid hormones, when present in excess, affect the cardiovascular (CV) system by increasing heart rate, myocardial contractility, left ventricular mass, and the predisposition to supraventricular arrhythmias (74). Lipophilic T3 binds to the thyroid hormone receptor (TR) upon entry into the cardiomyocyte nucleus. Activation of TR results in stimulating gene transcription of the heavy alpha chain of myosin, calcium ATPase, Na/K-ATPase, beta 1 adrenergic receptor, and atrial natriuretic peptide (75–77). Genomic and non-genomic effects of thyroid hormones on cardiomyocytes lead to increased myocardial contractility, which, among other hemodynamic effects, results in increased heart rate, increased circulating volume, left ventricular volume, ejection fraction, and cardiac output (74).

Subclinical thyroidopathies can harm the cardiovascular system and manifest as an increase in CV morbidity and mortality by 20-80% (78, 79). Regardless of whether it is of endogenous or exogenous origin, in subclinical hyperthyroidism type 2 (TSH <0.1mIU/ml), the risk of AF presence is higher (HR 2.54 *vs.* 1.63) than in type 1 (TSH 0.1-0.4mIU/ml) (80, 81). Subclinical hyperthyroidism is associated with an increased left ventricular mass that increases ejection fraction (EF) and diastolic dysfunction (80, 82, 83). Clinical hyperthyroidism is associated with a 16% increased risk of major CV events commonly manifested as worsening heart failure, including high-output heart failure (84). It is noteworthy that an increased supraventricular ectopic activity often accompanies hyperthyroidism (85).

## 5 Pathophysiology of Cardiac Rhythm Disorders

Abnormal Ca^2+^ handling within cardiomyocytes is central to many types of arrhythmias. Arrhythmia-related contractions begin with external Ca^2+^ entering the cell’s cytosol through the L-type calcium channels to signal the sarcoplasmic reticulum to release more Ca^2+^ *via* ryanodine receptor channels (RyRs) - specifically the RyR2 isoform (86, 87). RyRs facilitate downstream calcium-dependent processes throughout the cell, e.g., actin-myosin contraction (88, 89). Inositol 1,4,5-trisphosphate receptor (IP3R) also responds to cellular cues to release Ca^2+^ from the SR (90). Ca^2+^ release from the SR initiates contraction in cardiac myocytes, but removing Ca^2+^ from the cytosol following systole is equally vital. In diastole, sarco/endoplasmic reticulum calcium ATPase translocates cytosolic Ca^2+^ back into the SR. Diastole is a sensitive time window for Ca^2+^ clearance, and improper Ca^2+^ clearance can have significant arrhythmogenic consequences (87). Mutations or covalent modifications of RyR channels can cause Ca^2+^ leakage across the SR membrane during diastole, promoting arrhythmias (91, 92). Our recent work shows that Zn^2+^ regulates the open probability of RyR2 in a manner that regulates beat-to-beat contractions in cardiomyocytes (93). This finding suggests that aberrant intracellular zinc homeostasis could contribute to arrhythmogenic events. Premature Ca^2+^ flux from the SR results in untimely depolarization events known as early afterdepolarizations (EADs) and delayed afterdepolarizations (DADs) (87). Ventricular arrhythmias can be caused by abnormal Ca^2+^ handling, electrolyte imbalance, and/or myocardial scarring (94). Physical barriers can also promote arrhythmia (e.g., fibrotic tissue) developing from ischemia and subsequent scarring (95, 96). Although myocardial scarring is the common feature in fatal ventricular arrhythmias, metabolic abnormalities may also play a significant role.

### 5.1 Cardiac Arrhythmias Associated With Thyroid Hormones

**Figure 2:**
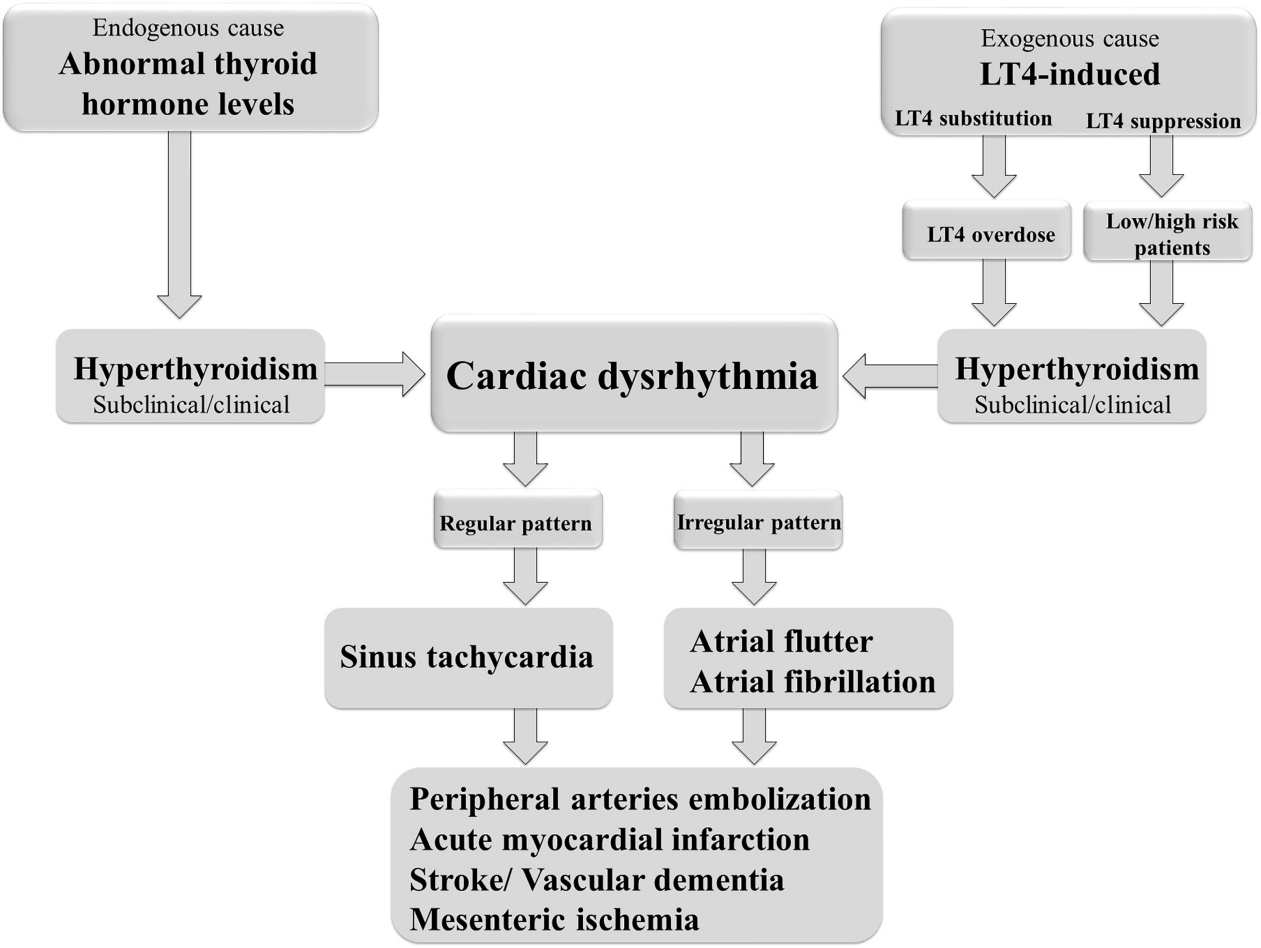
Thyroid dysfunction and the risk of cardiac arrhythmias.

Cardiac arrhythmias are defined as irregular heartbeats and range in severity. They are generally defined by the affected region of the heart and the type of defect: supraventricular arrhythmias (brady- and tachyarrhythmias including atrial premature complexes) and ventricular arrhythmias. AF is one of the most common types of chronic arrhythmia, recognized in an electrocardiogram as an irregular P–R interval and a missing P wave. AF occurs more frequently in obese people and is the most common arrhythmia associated with abnormal thyroid hormone levels (97). It is a highly prevalent arrhythmia promoting heart failure, embolic stroke, and death (98). Even short, subclinical episodes of AF are associated with an increased risk of stroke (99). Paroxysmal and sustained or permanent forms of AF confer a significant clinical burden and worsens the patient’s quality of life. AF is the most common cardiac complication of hyperthyroidism and LT4-induced thyrotoxicosis (97, 100) (**Figure 2**). Sinus tachycardia and atrial flutter are also commonly associated with hyperthyroidism (101). AF in thyrotoxicosis is associated with significant mortality and morbidity resulting from embolic events (97). The risk factors for AF in patients with hyperthyroidism are similar to those in the general population. They include age, male sex, and a history of ischemic, congestive, or valvular heart diseases (102).

AF occurs in up to 15% of patients with hyperthyroidism (103), compared with 4% in the general population (104). AF is more common in men and patients with T3 toxicosis (97). Also, subclinical hyperthyroidism is associated with an almost 3-fold increase in the risk of developing AF (103). Once initiated, AF alters the electrical and structural properties of the atria in a manner that affects its maintenance, increasing the risk of recurrence and can alter the response to antiarrhythmic drugs (97, 105). In addition, AF increases the risk of cerebrovascular stroke, peripheral embolization, and overall mortality (106–108). About 13-15% of individuals with newly developed AF have biochemical hyperthyroidism (103). The risk factors of developing AF in hyperthyroid individuals are age, pre-existing ischemic or valvular heart disease, or heart failure (85). Analysis of Framingham study results, related to the frequency of AF during a ten-year follow-up of patients >60 years, showed that AF occurred in 28% of those with subclinical hyperthyroidism, as opposed to 11% euthyroid patients. Furthermore, decreased TSH values, even with normal serum thyroid hormone values, were associated with a 3-fold increase in the frequency of AF (103). Heering et al. (Rotterdam study) showed a higher incidence of AF and sudden cardiac death in people >55 years with low normal TSH values and high normal FT4 levels (109). Studies using Mendeleev’s randomization to demonstrate an association between thyroid dysfunction and cardiovascular disease also reported an association between hyperthyroidism and AF (110, 111).

#### 5.1.1 LT4 Therapy and Cardiac Arrhythmias

Effects of T3 such as acceleration of cardiac depolarization and repolarization, the shortening of the action potential duration, and the refractory period of the atrial myocardium and AV node are not observed in mono-LT4 therapy. Namely, the production of T3 in patients on LT4 substitution, primarily after thyroidectomy, is related to the peripheral deiodination of T4. Antithyroid therapy and beta-blockers affect heart rate control, even conversion to normal sinus rhythm in about 60% of patients (85, 112, 113). The most important factor influencing the conversion of AF to sinus rhythm in hyperthyroidism is the duration of AF (114). In cases where AF lasts for more than a year in the elderly and is resistant to antithyroid and beta-blocker therapy, AF is often associated with ischemic heart disease (115). In the cases where LT4 use (exogenous hyperthyroidism) induces TSH suppression, incidences of CV and arrhythmic events are increased compared to the general population (52). AF, provoked either by endogenous or exogenous hyperthyroidism, results in a significant increase in overall morbidity and mortality, mainly caused by the consequences of systemic embolism (97). Compared to euthyroid subjects, patients with suppressed TSH have increased sympathetic autonomic activity and decreased parasympathetic tone, resulting in increased heart rate variability and prolonged QT interval (116). The mentioned changes in the CV system in patients with subclinical or clinical hyperthyroidism may result in an increased frequency of cardiac arrhythmias, primarily AF (106), and a higher frequency of systolic and diastolic left ventricular dysfunction (74, 117).

In addition to AF, in patients on LT4 suppressive therapy, sinus tachycardia and shortening of the PR interval are often detected electrocardiographically as manifestations of accelerated atrioventricular conduction (118–120). The prolonged P wave is most often seen as a manifestation of impaired interatrial conduction, while a delay in intraventricular conduction often results in a right bundle branch block (121). Ventricular arrhythmias in patients on LT4 suppressive therapy are rare, and their presence should always arouse suspicion of pre-existing heart disease. It should be noted that the incidence of ventricular fibrillation (VF) attributed solely to the thyroid status imbalance is less frequent in humans compared to experimental animals, which are prone to both AF and VF in response to an excess of thyroid hormones (TH) (101, 122, 123).

Epidemiological studies have shown higher cardiovascular and all-cause mortality in patients with endogenous and exogenous hyperthyroidism than in the general population (55, 124, 125). Also, a study by Klein-Hesselink et al. reports 3.3 times higher mortality due to CV in patients with differentiated thyroid cancer (DTC). In comparison, mortality from all causes was 4.4 times higher than in the general population, regardless of age, gender, and cardiovascular risk factors. Furthermore, each 10-fold decrease in TSH levels increased the risk of cardiovascular death by 3-fold (126). A study by Suha et al. showed similar results, demonstrating a higher incidence of coronary heart disease (CVD) and cerebrovascular insult (CVI) in patients diagnosed with DTC, with the risk of CVD and CVI being directly proportional to the dose of LT4 administered (127).

Although the use of suppressive doses of LT4 can lead to a significant reduction in TN volume and diffuse atoxic goiter, the potential side effects of this therapy on the CV system and bone metabolism, especially in people over 60 years and postmenopausal women, are limiting factors for the routine use of this therapeutic approach (48). However, using supraphysiological LT4 doses in younger patients to suppress TSH levels reduces TN volume in one out of six patients without significant comorbidity (128).

#### 5.1.2 LT4 Combined With T3 Therapy and Cardiac Arrhythmias

The combined use of LT4 and LT3 has multiplied over the last decade. The impression is that such increases in use are rarely observed in the group of patients that strictly require combination therapy, but mainly result from patients wishing to have experience with its use or pharmaceutical companies’ pressure that favors its use in patients unsatisfied with LT4 treatment alone. The rationale for using T4 and T3 combination therapy is that defects in deiodinase enzymes that convert thyroxine to triiodothyronine could lead to persistent symptoms in patients despite being biochemically well-regulated on LT4 monotherapy (129). A lack of more extensive studies pointed out LT3+LT4 positive effects on patients’ wellbeing and the increase in the previously insufficient functional capacity of peripheral tissues. The real benefit of the LT3+LT4 combination could be expected in a relatively small number of hypothyroid patients. ETA suggests the LT3+LT4 combination as an experimental 3-month trial observed by an experienced endocrinologist in LT4 well-compliant patients persistently presented with hypothyroidism-associated complaints, despite normal TSH levels. If there is no improvement in LT3+LT4 treated patients after 3 months of use, it should be discontinued (130). LT3+LT4 combination is not recommended in patients with cardiac arrhythmias, as increased free T3 could act pro-arrythmically in prone patients (130, 131).

Additionally, Regalbuto et al. did not show any advantage of combined LT3+LT4 over LT4 monotherapy suppression in totally thyroidectomized patients for thyroid cancer regarding improved wellbeing and peripheral tissue response. Even though thyroid function tests suggested subclinical hyperthyroidism, the clinical syndrome of LT3 and LT4 excess was not registered (132). Similarly, Tariq et al. did not find any additional risk for atrial fibrillation and cardiovascular disease in patients on combined LT3+LT4 therapy (133).

## 6 Cardiac Electrical Remodeling Associated With Hyperthyroidism and LT4 Treatment

Electrophysiological studies reveal that TH modifies f-channel conductance in sinoatrial cells, which changes the diastolic depolarization rate (134–136). This finding suggests a direct effect on myocardial membrane-related electrogenesis. Moreover, it provides the potential mechanism behind bradycardia and sinus tachycardia’s association with hyperthyroidism.

Cardiac arrhythmia classification assumes disturbance of rhythm results from abnormal 1) impulse initiation and/or 2) intercellular impulse propagation (137). The abnormal impulse initiation is associated with abnormal automaticity and/or a triggered activity (induced by EAD or DAD). On the other hand, abnormal intercellular impulse propagation refers to a block of conduction and re-entry. Re-entry occurs when the propagating impulse persists due to continuous activity, after normal activation of the heart, instead of dying out, that re-excites the heart after the refractory period has ended (138).

TH effects on the development of AF and VF are more complex than chronotropic effects. There are several potential mechanisms by which TH can trigger arrhythmicity in the heart. Such effects are likely exerted through the same mechanisms as hyperthyroidism. One such mechanism is its direct involvement in controlling the transcription of genes encoding ion channels and other proteins involved in signal transduction. For example, TH regulates mRNA transcription of voltage-activated K^+^ channel genes, including those encoding Kv4.3, Kv.4.2 (which contribute to the transient outward potassium current) and Kv1.4, Kv1.5, and Kv1.2 (which contribute to the ultra-rapid delayed rectifier potassium current) (139–143), and about nine ion channel α- and β-subunits (144). Hyperthyroidism was found to up-regulate the expression of Kv1.5 mRNA, particularly in the atrium of the heart (145). Sunagawa et al. demonstrated decreased L-type Ca^2+^ channel expression in the atria and Kv1.2 and Kv1.4 in both atrial and ventricular tissue in LT4-treated rats (136). Interestingly, despite this decrease in L-type Ca^2+^ channel expression, the L-type Ca^2+^ current increased (146–148), most likely due to the TH-induced transcriptional regulation of myocardial Ca^2+^ cycling proteins.

TH has the potential to influence Ca^2+^ levels in cardiomyocytes through multiple mechanisms, including regulating the expression of sarcoplasmic reticulum Ca^2+^-ATPase (SERCA2) and RyR2 [Ca^2+^ cycling proteins; (149)], and down-regulating Na^+^/Ca^2+^ exchanger and phospholamban (150–153). Increased Ca^2+^ influx and efflux rates also characterize hyperthyroidism in ventricular cells (154) most likely due to altered activation of sarcolemmal Ca^2+^ channels and SERCA2 activity (148, 155). There are also rapid non-genomic TH responses that modulate the activity of Ca^2+^ cycling proteins and have consequent effects on intracellular Ca^2+^ currents (147, 148, 150, 156). It is expected that LT4 therapy can induce aberrant Ca^2+^ homeostasis through altered Ca^2+^ handling similar to hyperthyroidism/TH activity.

Another mechanism by which clinical use of LT4 may trigger arrhythmia is the modulation of cardiac connexins. Connexin-43 (Cx43) is expressed in the ventricles and atria of the heart and is responsible for gap junction formation and thus the transmission of electrical signals between cells. TH receptors bind to the Cx43 promoter, indicating that TH can modify the expression of Cx43 mRNA synthesis (157). For example, it has been shown that Cx43 protein levels increase in TH-treated neonatal cultured cardiomyocytes (158). Also, in the atria and ventricles of hyperthyroid rats, phosphorylation of Cx43 isoforms is reduced, compared to untreated controls (159, 160). This is interesting as VF (159) and AF (101) susceptibility increases in T3-treated rats when myocardial Cx43 phosphorylation decreases. Also, TH-treated rat liver epithelial cells stimulate gap junctional communication and Cx43 mRNA expression (161).

## 7 Conclusions

Patients who have undergone thyroidectomy exhibit compromised production of thyroid hormones, which warrant hormone replacement therapy to avoid developing hypothyroidism. To date, the standard treatment in such instances is a long-term LT4 treatment regimen. LT4 treatment after thyroidectomy to treat cancer has the added advantage of suppressing the local and distant malignancy from spreading. However, each patient’s LT4 dose must be optimized to avoid potential side effects such as weight loss, sweating, anxiety, insomnia, osteoporosis (increased bone fracture risk), and an increased heart rate. Thus, LT4 treatment is not recommended in recent heart attack patients.

Nonetheless, patients with no known CV issues can experience CV complications following LT4 treatment, which is not surprising given the role of TH in regulating cardiac functioning. It is associated with an increased risk of developing heart arrhythmias, primarily AF, as well as osteoporosis. Although, studies do show that LT4 treated patients with TSH levels from 0.04 to 0.4 mIU/ml had not experienced an increased risk of cardiovascular disease, arrhythmias, or osteoporotic fracture. Also, LT4 doses in younger patients to suppress TSH levels reduce TN volume in one out of six patients without significant comorbidities. Nevertheless, suppressive LT4 doses can lead to potential side effects of this therapy on the CV system and bone metabolism, especially in people over 60 years and postmenopausal women. Thus, there are limiting factors for the routine use of this therapeutic approach, but improving the approach to individualized medicine in the at-risk population (implementing less excessive LT4 treatment regimens and monitoring more LT4 arrhythmia triggers) may reduce cardiac arrhythmia risk and mortality.

## Author Contributions

ZG, MO, and EI designed, wrote and supervised the manuscript. AJS, ME, SJP, VS, and SS wrote the manuscript. AJS and TG critically revised the manuscript. All authors contributed to manuscript revision, and approved the submitted version.

## Funding

This work was funded by the Ministry of Education, Science and Technological Development of the Republic of Serbia (Contract No#451-03-9/2021-14/200017) and KAUST grant OSR#4129 (awarded to EI and VBB), which also supported MO. ME has been supported by the KAUST Office of Sponsored Research (OSR) Award no. FCC/1/1976-17-01, and TG by the King Abdullah University of Science and Technology (KAUST) Base Research Fund (BAS/1/1059-01-01).

## Conflict of Interest

The authors declare that the research was conducted in the absence of any commercial or financial relationships that could be construed as a potential conflict of interest.

## Publisher’s Note

All claims expressed in this article are solely those of the authors and do not necessarily represent those of their affiliated organizations, or those of the publisher, the editors and the reviewers. Any product that may be evaluated in this article, or claim that may be made by its manufacturer, is not guaranteed or endorsed by the publisher.
